# Identification of putative novel O-glycosylations in the NK killer receptor Ncr1 essential for its activity

**DOI:** 10.1038/celldisc.2015.36

**Published:** 2015-12-22

**Authors:** Ariella Glasner, Ziv Roth, Alexander Varvak, Antonija Miletic, Batya Isaacson, Yotam Bar-On, Stipan Jonjic, Isam Khalaila, Ofer Mandelboim

**Affiliations:** 1 The Lautenberg Center for General and Tumor Immunology, The Hebrew University Hadassah Medical School, The Institute of Medical Research Israel Canada (IMRIC), Jerusalem, Israel; 2 Department of Life Sciences and the National Institute for Biotechnology in the Negev, Ben-Gurion University of the Negev, Beer-Sheva, Israel; 3 The Mina and Everard Goodman Faculty of Life Sciences, Bar-Ilan University, Ramat Gan, Israel; 4 Department of Histology and Embryology Center for Proteomics, Faculty of Medicine, University of Rijeka, B. Branchetta, Rijeka, Croatia; 5 Avram and Stella Goldstein-Goren Department of Biotechnology Engineering, Ben-Gurion University of the Negev, Beer-Sheva, Israel

## Abstract

Natural killer (NK) cells kill tumor and virus-infected cells using activating NK cell receptors. One of the major NK-activating receptors is NKp46 and its mouse ortholog Ncr1. NKp46/Ncr1 is expressed exclusively on NK cells and on a subset of innate lymphoid cells. NKp46/Ncr1 was shown to be involved in a myriad of pathologies and immunological settings. Specifically, NKp46/Ncr1 was shown to interact with the viral hemagglutinin (HA) protein and with an unknown tumor/cellular ligand. NKp46 and Ncr1 are structurally similar; however, they are substantially different in their glycosylation patterns. Although the human NKp46 carries both O- and N-glycosylations that are essential for its activity, the mouse Ncr1 was predicted to have N-linked glycosylations only. Here we discovered using prediction algorithms and high-performance liquid chromatography analysis that Ncr1 carries two putative novel O-glycosylations, one of which (Thr 225) is conserved in NKp46. We next used surface plasmon resonance, biochemical, mutational and functional *in vitro* and *in vivo* assays to demonstrate that the putative O-glycosylations of Ncr1 are critical for its function.

## Introduction

Nature killer (NK) cells are lymphocytes of the innate immune system. They recognize and eliminate virally infected cells [1–3], various malignancies [4–7], self-cells [8, 9] and bacteria [10–12]. NK cell cytotoxicity is executed by various activating receptors such as NKG2D, 2B4, NKp80 and the natural cytotoxicity receptors (NCRs): NKp30, NKp44 and NKp46 [13]. Of the NCRs, only NKp46 has a functional mouse Ortholog, named Ncr1 [14]. The importance of NK cells in general, and NKp46/Ncr1 in particular was well demonstrated, primarily via the use of Ncr1 knockout mice, denoted Ncr1^*gfp*/*gfp*^ [2, 4, 5, 7, 11, 12, 15–18]. However, despite years of research, the identity of the cellular/tumor ligand/s recognized by NKp46/Ncr1 is still obscure. In contrast, the hemagglutinin (HA) proteins of influenza virus and Sendai as well as the HA proteins of Newcastle [19] and Pox viruses [3] were discovered as ligands for the activating receptors NKp46/Ncr1 and NKp44. Sialic acids attached to glycosylated residues of NKp44 and NKp46 were shown to be critical for their interaction with HA [1, 18, 20]. Although very similar in structure, the human NKp46 and the murine Ncr1 differ in their glycosylation pattern. Whereas the human NKp46 is predicted to have two O-glycosylated residues: Thr 125 and Thr 225 and one N-glycosylated residue: Asn 216 [21], the murine Ncr1 is predicted to have three N-glycosylations, expressed on Asn 139, Asn 216 and Asn 238 and no O-glycosylated residues [14]. Interestingly, the O-glycosylated residue Thr 225 was shown to be critical for the human NKp46 activity against pathogens such as influenza and for the recognition of various tumors [1]. Since the mouse Ncr1 was not predicted to have O-glycosylated residues, the question is raised whether the mouse Ncr1 accurately mimics the human NKp46 activities and whether NKp46 function against tumors and pathogens can be studied in mice. Here we discovered that Ncr1 expresses two, putative previously unknown O-glycosylated residues essential for its function, one of which, Thr 225, is conserved.

## Results

### Identification of novel O-linked glycosylated residues in Ncr1

**Figure 1:**
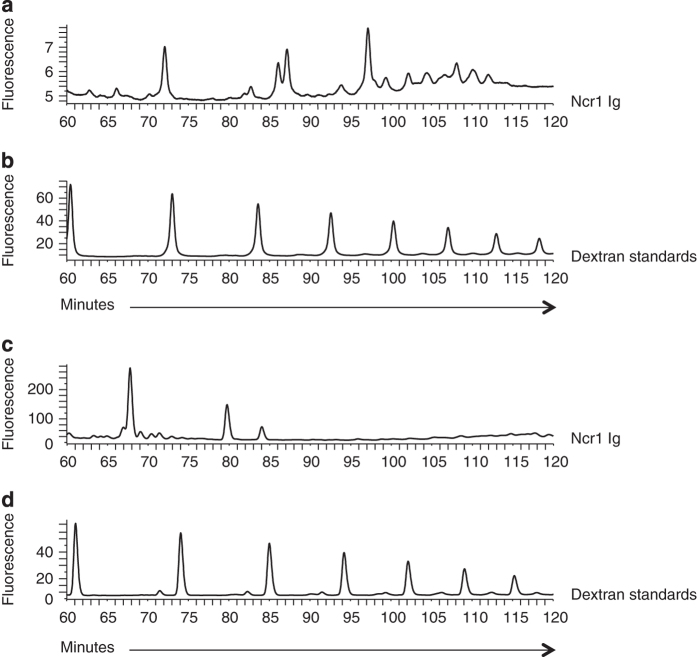
Characterization of the Ncr1 glycosylations. (**a**–**d**) HPLC chromatogram of N- (**a**) and O- (**c**) linked glycan release from the Ncr1 Ig fusion protein. The Ncr1 Ig fusion proteins N-linked glycans were released in solution with PNGase F before O-linked glycan analysis. (**b**, **d**) are the dextran standards. The data are shown as fluorescence arbitrary units. The HPLC chromatograms are representatives of two independent runs. The chromatogram shown in **a** is identical to a figure in a different manuscript (Glasner *et al.* JI 2015, in press), as it served as control in experiments pertaining to both papers, performed simultaneously.

Ncr1 is predicted to have three N-linked glycosylations at positions N139, N216 and N238 [14]. Because the O-glycosylations of NKp46 were shown to be critical for its recognition of viral HA [1] and as Ncr1 also recognizes viral HA [18], we predicted that Ncr1 carries additional, yet unidentified O-glycosylations. To identify these potential glycosylated residues, we used the NetOGlyc 4.0 Server for mucin type O-GalNAc O-glycosylation [22] and predicted the existence of two putative O-glycosylated residues at positions Thr 222 and Thr 225 (both located in the Ncr1 stalk domain; Supplementary Figure S1). To investigate whether Ncr1 indeed carries O-glycosylated residues, we initially generated an Ncr1 Ig fusion protein composed of the extracellular part of Ncr1 fused to human IgG1. Then, we subjected the Ncr1 Ig fusion protein to N-glycan and O-glycan release by hydrazinolysis. As expected, the high-performance liquid chromatography (HPLC) chromatogram of the N-glycosylations showed three prominent peaks (Figure 1a), indicating the existence of N-linked glycosylations in the Ncr1 fusion proteins. The dextran standards are shown in Figure 1b. As predicted (Supplementary Figure S1), two prominent peaks that were eluted from the column at ~70 and ~80 min were clearly evident in the Ncr1 Ig subjected to O-glycan release (Figure 1c), indicating that Ncr1 Ig also carries previously unidentified O-glycosylations. The dextran standards are shown in Figure 1d. The O-glycosylation analysis was performed following the removal of N-glycosylation by PNGase F (see Materials and Methods).

### Ncr1 isolated from primary NK cells carries O-linked glycosylations

**Figure 2:**
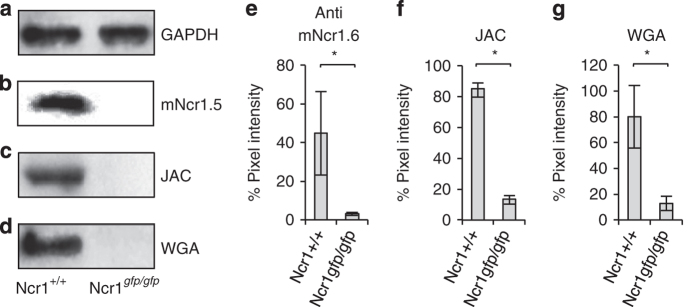
Ncr1 expressed on primary NK cells carries O-linked glycosylations. (**a**) Western blot (WB) for GAPDH in the whole-cell lysates of purified NK cells derived from WT and Ncr1^*gfp/gfp*^ mice before imunoprecipitation. (**b**–**d**) Ncr1 protein from WT and Ncr1^*gfp/gfp*^ mice was immunoprecipitated with the mNcr1.6 mAb, blotted and stained with the mNcr1.5 mAb (**b**), Jacalin (JAC) (**c**) and wheat germ agglutinin (WGA) (**d**) lectins. WB figures were adjusted for better clarity. (**e**–**g**) Ratio between the GAPDH staining and the mNcr1.5 mAb (**e**) or lectins (**f**, **g**) staining (quantified by pixel intensity). Values are shown as mean±s.e.m.; *P<0.05. The figures combine three independent experiments.

To demonstrate that Ncr1 expressed on primary mouse NK cells is O-glycosylated, we initially immunoprecipitated the Ncr1 protein from NK cells obtained from wild-type (WT) (Ncr1^*+/+*^) and (Ncr1 knockout Ncr1^*gfp/gfp*^) mice [2]. GAPDH levels in the cell lysates were more or less equivalent (Figure 2a). As can be seen in Figure 2b, Ncr1 was successfully precipitated (detected by the anti-Ncr1 monoclonal antibody (mAb) that we have generated, mNcr1.5). The Ncr1 knockout mice do not express the protein. Next, we stained the precipitated proteins with lectins that preferentially bind O-linked glycosylations: Jacalin (JAC) [23] and wheat germ agglutinin [24]. As can be seen in Figure 2c and d, both lectins recognized the endogenous mouse Ncr1 protein, suggesting that Ncr1 carries putative O-linked glycosylations. The results were quantified in Figure 2e–g.

### Residues T222 and T225 of Ncr1 are O-glycosylated

To demonstrate that the predicted T222 and T225 residues of Ncr1 are indeed O-glycosylated, we mutated each of the predicted glycosylated threonine (Thr) residues separately to alanine (Ncr1 T222A and Ncr1 T225A) and both Thr residues together (Ncr1 T222 225A). We fused the extracellular parts of these receptors to the Fc part of human IgG1 and produced the corresponding fusion proteins.

It is very unlikely that mutating the O-glycosylated residues T222 and T225 of Ncr1 would affect the protein structure. This is because both residues are located in the stalk domain of Ncr1. Although the crystal structure of Ncr1 was not solved yet, the structures of NKp46 and NKp30 were solved [25–28] and in both cases, the stalk domains were unstructured, indicating that the stalk is highly flexible and does not adopt a defined secondary structure. Because the structures of Ncr1 and NKp46 are very similar, composing of a stalk domain and two Ig domains (Supplementary Figure S1), it is very probable that the stalk domain of Ncr1 that contains the O-glycosylated residues, does not adopt a defined secondary structure either. Nevertheless, to exclude the possibility that the mutations in the Ncr1 Thr residues disrupted somehow the protein structure, we generated eight new anti-Ncr1 mAbs and tested whether the various WT and mutated Ncr1 fusion proteins can be recognized by the various mAbs. All eight mAbs recognized all WT and mutated fusion proteins equally well (Supplementary Figure S2), suggesting that the folding of the mutated Ncr1 fusion proteins was not disrupted.

**Figure 3:**
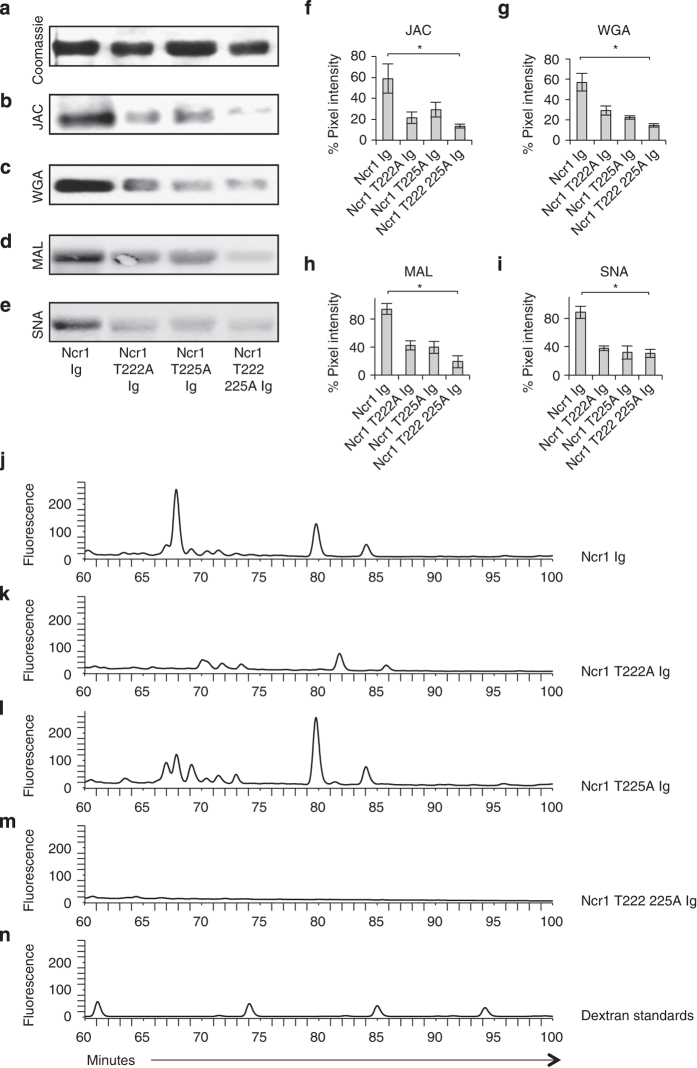
Thr 222 and Thr 225 are glycosylated. (**a**) Coomassie staining of the various Ncr1 fusion proteins (5 μg) run on 10% SDS-PAGE gel under reducing conditions. (**b**–**e**) Western blot (WB) performed on the various Ncr1 fusion proteins shown in **a**. Staining was performed with Jacalin (JAC) (**b**), wheat germ agglutinin (WGA) (**c**), Maackia amurensis lectin II (MAL) (**d**) or Sambucus nigra (SNA) (**e**) lectins. WB and Coomassie figures were adjusted for better clarity. The figures combine at least three independent experiments. (**f**–**i**) Ratio between the Coomassie staining and the WB staining (quantified by pixel intensity) of the various lectins. Values are shown as mean±s.e.m.; *P<0.05. (**j**–**n**) HPLC chromatogram of O-linked glycan release from the Ncr1 Ig (**j**), Ncr1 T222A Ig (**k**), Ncr1 T225A Ig (**l**) and Ncr1 T222 225A Ig (**m**) fusion proteins and dextran standards (**n**). The various Ncr1 Ig fusion proteins N-linked glycans were released in solution with PNGase F before O-linked glycan analysis. The data are shown as fluorescence arbitrary units. The chromatogram shown in **j** is identical to the one shown in Figure 1c albeit presented in a different time scale. The HPLC chromatograms are representatives of two independent runs.

Next, we ran the various Ncr1 Ig proteins on SDS-polyacrylamide gel electrophoresis (SDS-PAGE) gels (Figure 3a shows the Coomassie staining) and performed western blot assays with four lectins: Jacalin (Figure 3b), wheat germ agglutinin (Figure 3c) and two additional lectins, Maackia amurensis lectin II, which preferentially binds sialic acid residues attached in an α2,3 linkage (Figure 3d) and the Sambucus nigra lectin, which preferentially binds sialic acids attached to a terminal galactose in an α2,6 linkage [29] (Figure 3e). The WT Ncr1 was well stained by all lectins, indicating that the novel O-linked glycosylations are present in the fusion proteins. Importantly, each of the mutated fusion proteins was stained to a significantly lower degree by all lectins (Figure 3b–e, quantified in Figure 3f–i). To directly demonstrate that residues 222 and 225 carry O-linked glycosylations, we subjected each of the WT and mutated fusion proteins to O-glycan release by hydrazinolysis and compared the O-linked glycosylations profiles following HPLC separations. The O-glycosylation analysis was performed following the removal of N-glycosylations by PNGase F (see Materials and Methods). As can be seen in Figure 3j–m, both prominent peaks, observed in the WT Ncr1 at ~70 and ~80 min (Figure 3j), were significantly reduced in the Ncr1 T222A Ig (Figure 3k). In the Ncr1 T225A Ig (Figure 3l), only the ~70 peak was reduced and in the double mutated Ncr1 T222 225A Ig (Figure 3m), both peaks disappeared. The dextran standards are shown in Figure 3n. We concluded that probably, in the WT Ncr1 there are three major glycoforms, which occupy both T222 and T225. In the T222A mutant there is a decrease in the ~80 and ~85 peaks and disappearance of the ~70 peak, which might be explained by the loss of the T222A site. In the T225A, the increase in the ~80 peak and the decrease in the ~70 peak might indicate a sugar composition change in which the occurrence of the ~80 peak glycoform in position T222 is favorable.

### The O-linked, but not the N-linked residues of Ncr1 are important for its recognition and function against various tumors

**Figure 4:**
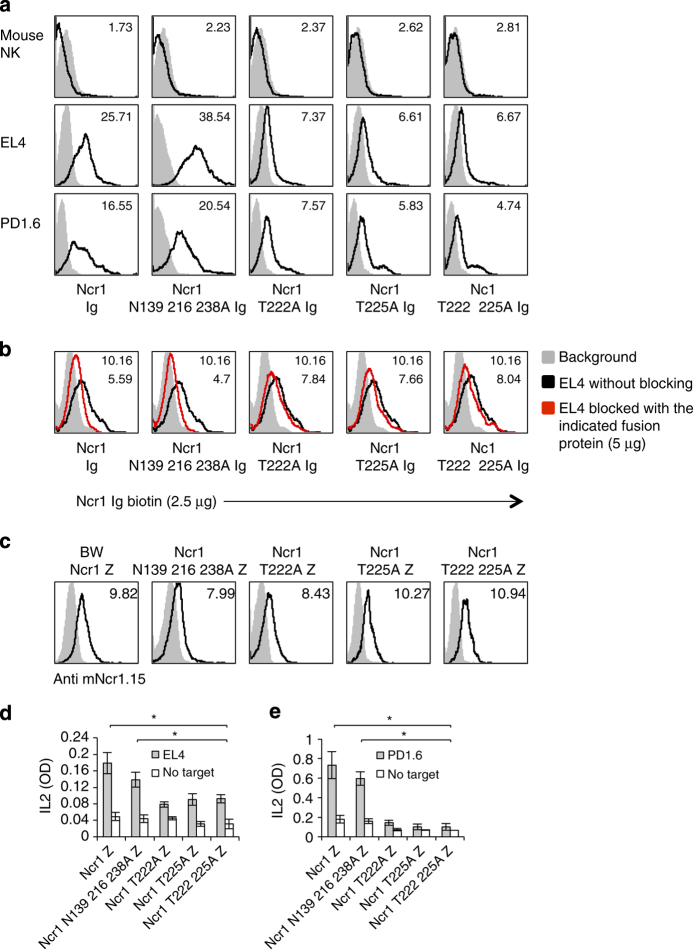
The O-linked glycosylations of Ncr1 are important for its function against various tumors. (**a**) FACS staining of murine cell lines and primary NK cells, as indicated. Staining was performed with various Ncr1 Ig fusion proteins (listed in the bottom of the figure, black line histograms). The gray filled histograms are the backgrounds secondary mAb staining. The median fluorescent intensities (MFIs) of the fusion proteins staining are indicated. (**b**) FACS staining of EL4 cells that were untreated or blocked with the indicated fusion proteins. The cells were then stained with biotinilated Ncr1 Ig. The gray filled histograms are the backgrounds secondary mAb staining of the unblocked EL4 cells. The blocked EL4 cells background was identical to the unblocked and is not shown in the figure. The MFIs of the fusion proteins staining are indicated. (**c**) FACS staining of BW transfectants expressing the various Ncr1 proteins, as indicated. Staining was performed with anti-mNcr1.15 mAb (black line histograms). The gray filled histograms represent the background staining with the secondary anti-mouse antibody. MFIs of the anti-Ncr1 mAb staining are indicated. (**d**, **e**) IL2 secretion from the various BW transfectants presented in (**c**) following 48-h incubation with murine EL4 (**d**) and PD1.6 (**e**) cells. Values are shown as mean ±s.e.m. *P<0.05. The entire figure represents data collected from three independent experiments of each assay.

The identity of the tumor ligand/s of NKp46/Ncr1 is still unknown. To gain insight into the molecular properties of the NKp46/Ncr1 unknown tumor ligand and to test whether the O- and N-glycosylations of Ncr1 are involved in its tumor recognition, we prepared an additional Ncr1 fusion protein, in which all N-linked glycosylated residues are mutated (Ncr1 N139 216 238A Ig). Similarly to the O-glycosylations mutants, it seems that mutating the N-glycosylated residues of Ncr1 did not affect its folding as all eight anti-Ncr1 mAbs recognized this fusion protein (Supplementary Figure S2). Next, we assayed the binding of the various mutated Ncr1 Ig proteins to murine tumors. As can be seen in Figure 4a, the negative control, primary mouse NK cells, were not recognized by any of the tested fusion proteins. The EL4 and PD1.6 mouse cell lines were recognized by Ncr1 Ig and slightly better by the Ncr1 N139 216 238 Ig. Importantly, recognition by the O-linked mutants of Ncr1 was markedly impaired (Figure 4a). Similar results were obtained with various other mouse and human tumor lines (Supplementary Figure S3A and B, respectively). Thus, we concluded that the O-linked residues of Ncr1 have an important role in its tumor ligand recognition.

To further confirm the above results, we also performed competition assays. In these experiments, we incubated EL4 cells with 5 μg (saturating concentration) of each of the various WT and mutated Ncr1 fusion proteins. Next, we used a biotinilated Ncr1 Ig to stain the blocked and unblocked EL4 cells. Before these assays, we confirmed that the binding of the biotinilated and non-biotinilated Ncr1 Ig fusion proteins is identical (data not shown). As can be seen in Figure 4b, preincubation of EL4 cells with the WT Ncr1 Ig or with the Ncr1 N139 216 238A Ig mutant blocked the biotinilated Ncr1 Ig binding. In contrast, little or no blocking was observed with each of the O-linked mutated Ncr1 Ig fusion proteins (Figure 4b).

To test whether the newly identified O-linked Thr residues of Ncr1 are important for its function against tumors, we used a cell-based reporter assay in which we cloned the extracellular parts of the mouse Ncr1 and its various N- and O-sugar mutants, fused them to the mouse zeta chain and expressed them in mouse BW cells. In the BW reporter system, the triggering of Ncr1 leads to IL2 secretion, thus reporting for the functional interaction of Ncr1 with its ligand.

All mutated O-glycosylated proteins were expressed on the cell surface (Figure 4c), further indicating that the mutations did not affect the protein structure. In contrast, the expression of the triple N-mutant (Ncr1 N139 216 238A) was unstable. It appeared on the cell surface in delay and its expression was lost from the cell surface within a week.

To study the causes responsible for the unstable properties of the triple N-mutant Ncr1 (Ncr1 N139 216 238A), we expressed the full-length WT and mutated Ncr1 proteins in HEK 293T cells. We observed that while the WT and O-linked mutants were well expressed, the Ncr1 N139 216 238A appeared on the cells surface in delay and its expression was unstable due to endoplasmic reticulam arrest (data not shown). Because the expression of Ncr1 N139 216 238A zeta was unstable, experiments had to be performed quickly and be completed within a week. Incubation of WT Ncr1, as well as the Ncr1 N139 216 238A zeta mutant with EL4 and PD1.6 resulted in comparable levels of IL2 secretion (Figure 4d and e, respectively). In contrast, significantly less IL2 was secreted on incubation of EL4 or PD1.6 with all of the O-glycosylated mutants (Figure 4d and e). Thus, we concluded that the novel O-linked glycosylations of Ncr1 are important for its recognition of—and activation by—tumor ligands.

### The novel O-linked glycosylations of Ncr1 are essential for influenza recognition

**Figure 5:**
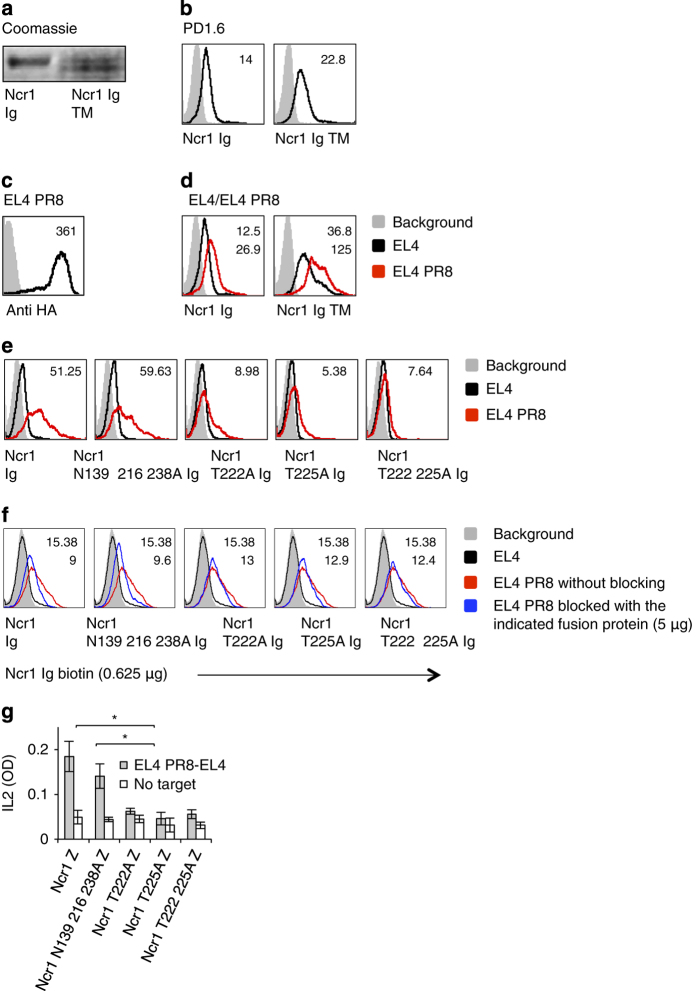
The O-linked glycosylations of Ncr1 are important for influenza virus HA recognition and function. (**a**) Coomassie staining of the indicated fusion proteins (5 μg) run on 10% SDS-PAGE gel under reducing conditions. Ncr1 Ig grown in medium containing tunicamycin is denoted Ncr1 Ig TM. Coomassie staining were adjusted for better clarity. (**b**) FACS staining of PD1.6 cells with Ncr1 Ig or with Ncr1 Ig TM. (**c**) Staining of PR8-treated EL4 cells with anti-HA1 mAb. (**d**) Untreated (black line histograms) or PR8-treated EL4 cells (red line histograms) were stained with Ncr1 Ig or with Ncr1 Ig TM (**e**). Untreated or PR8-treated EL4 cells were stained with each of the indicated WT or mutated Ncr1 Ig fusion proteins. (**f**) FACS staining of EL4 cells that were untreated or blocked with the indicated fusion proteins. The cells were then stained with biotinylated Ncr1 Ig. For all the FACS plots, the gray filled histograms are the backgrounds secondary mAb staining. The blocked or PR8-treated EL4 cells backgrounds were identical to the untreated cells therefore only the untreated cells background is shown). For all FACS plots, median fluorescent intensities (MFIs) of the fusion proteins staining are indicated. (**g**) IL2 levels secreted following incubation with the PR8-treated EL4 cells after subtraction of the untreated EL4 cells. Values are shown as mean ±s.e.m. *P<0.05. The entire figure represents data collected from three independent experiments of each assay.

To further corroborate the above results, we grew the Ncr1 Ig protein in the absence or presence of tunicamycin (TM), which blocks N-linked glycosylations in eukaryotic cells and ran the Ncr1 fusion proteins on SDS-PAGE gel. As can be seen in Figure 5a, the TM-treated Ncr1 Ig demonstrated a two-band pattern, which was different from the untreated Ncr1 Ig, suggesting that the TM treatment was effective. We next tested the binding of the TM-treated and untreated fusion proteins to the PD1.6 cell line and observed a slight increase in the binding of TM-treated Ncr1 Ig (Figure 5b). Similarly to what we observed with the Ncr1 N139 216 238A Ig (Figure 4a and Supplementary Figure S3). Similar results were obtained with other tumors (data not shown).

We have previously demonstrated that the Ncr1 glycosylations are important for its interaction with viral HA, although the identity of the glycosylated residues remained unknown [18]. Thus, to test whether the TM-treated Ncr1 Ig protein would still bind to influenza, we incubated EL4 cells with PR8 influenza (Figure 5c) and tested the staining with both Ncr1 proteins (TM treated or not). Significant elevation of staining by both proteins was detected (Figure 5d). Collectively, these data suggest that the N-glycosylations of Ncr1 are not important for its binding to influenza and tumor cells.

We next investigated whether the newly identified O-glycosylated residues of Ncr1 are important for its HA recognition. EL4 cells express high levels of the unknown Ncr1 tumor ligand (see figures above). Therefore, to minimize the background staining of Ncr1 Ig to its unknown tumor ligand/s, we used a low fusion protein concentration in which almost no Ncr1 Ig staining was observed (Figure 5e). Interestingly, although increased binding of the WT Ncr1 Ig and Ncr1 N139 216 238A Ig was observed to the PR8-treated cells (Figure 5e), the interaction of each of the O-linked mutated Ncr1 Ig fusion proteins with the PR8-EL4 cells was significantly less pronounced.

To further confirm the above results, we incubated EL4 cells with PR8 influenza and then preincubated the cells with 5 μg of each of the various WT and mutated Ncr1 Ig fusion proteins. Next, we stained the cells with the biotinilated Ncr1 Ig fusion protein. Pretreatment of PR8-EL4 cells with the WT Ncr1 Ig or Ncr1 N139 216 238A Ig blocked the biotinilated Ncr1 Ig binding. Pretreatment with each of the O-linked mutated Ncr1 Ig fusion proteins had little or no effect (Figure 5f).

We also tested the functionality of the O-glycosylated residues of Ncr1 against influenza by using the BW reporter system. Significantly, more IL2 was produced when the Ncr1 zeta and Ncr1 N139 216 238A zeta BW cells were incubated with EL4 PR8 influenza compared with the BW cells expressing the O-linked Ncr1 mutants (Figure 5g).

### The novel O-linked glycosylations of Ncr1 are important for its direct interaction with viral HA *in vitro* and *in vivo*

**Figure 6:**
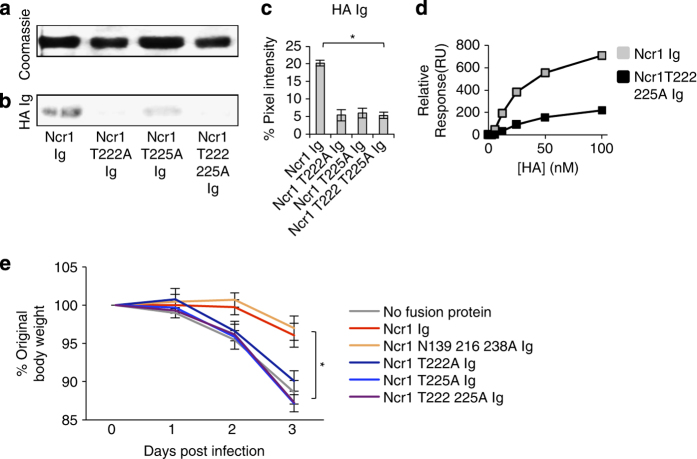
The O-linked glycosylations of Ncr1 are essential for its HA recognition and activation *in vitro* and *in vivo*.(**a**) Coomassie staining of the various fusion proteins (5 μg) run on 10% SDS-PAGE gel under reducing conditions. The same protein gel was used as loading control for the blotting presented in Figure 3 and here and therefore the Coomassie staining presented in **a** is the same as the one presented in Figure 3a and is shown here again only for better clarity. (**b**) Staining was performed with biotinylated HA Ig. The figures are representative of three independent experiments. Backgrounds in the Coomassie and in the WB staining were adjusted for better clarity. (**c**) Ratio between the Coomassie staining and the HA Ig staining (quantified by pixel intensity) in three independent experiments. Values are shown as mean±s.e.m. *P<0.05. (**d**) Surface plasmon resonance plots of the Ncr1 fusion proteins binding to HA. (**e**) Ratio between the original body weight before infection (day 0) and on 3 consecutive days following infection with influenza virus PR8, untreated or treated with the various fusion proteins. The experiment was repeated twice and at least eight mice were used in each group in each experiment. The figure represents data from the two experiments combined. Values are shown as mean±s.e.m. *P<0.05.

To demonstrate that Ncr1 directly interacts with viral HA via its O-glycosylated residues, we cloned the extracellular part of the HA protein of PR8 influenza and fused it to human IgG1. We next ran the WT Ncr1 protein and the O-glycosylated mutants of Ncr1 on SDS-PAGE gel (Figure 6a) and WB them with HA Ig. A direct HA Ig binding was detected to Ncr1 Ig. All O-mutants of Ncr1 were less recognized (Figure 6b, quantified in Figure 6c).

We also performed surface plasmon resonance experiments using WT Ncr1 Ig and Ncr1 T222 225A Ig immobilized on a CM5 sensor chip and the recombinant HA as analyte at a series of concentrations. Immobilizing the HA to the chip and passing Ncr1 Ig and Ncr1 T222 225A Ig as analytes was also tried, however conjugated HA protein gave no response with analyte Ncr1 Ig proteins, presumably because HA multimers did not stay intact on immobilization. We assessed binding affinities using relative response from a region of the sensogram that is closest to the steady state. Saturating conditions were not reached even with high concentrations of HA, therefore, the steady-state-binding model could not be applied. Dissociation of HA was also excessively slow to be measured, precluding application of the kinetic model. Thus, binding levels for a given concentration were compared. Although quantitative evaluation of the binding affinity constants between HA and Ncr1 Ig and its mutant was not possible, a qualitative comparison could be made on the basis of the response levels as a function of HA (analyte) concentration. As can be seen in Figure 6d, the binding of Ncr1 T222 225A Ig to HA was significantly impaired, compared with the WT Ncr1 Ig. Thus, we concluded that the newly identified O-linked glycosylations of Ncr1 are critical for the direct Ncr1 recognition of HA.

To test the function of the O-linked glycosylated residues of Ncr1 *in vivo*, we infected C57BL/6 mice with PR8 influenza virus. The virus was either untreated or preincubated with the WT and the sugar mutated Ncr1 Ig fusion proteins. We hypothesized that if the fusion proteins interact with the HA protein, we would expect the infection to be reduced because the HA protein is important for virus entry into the cells. As can be seen in Figure 6e, mice that were infected with influenza virus preincubated with fusion proteins that are able to bind HA, that is, WT Ncr1 Ig and Ncr1 N139 216 238 Ig, lost significantly less weight than mice that were infected with influenza virus preincubated with the O-glycosylated mutated fusion proteins.

Collectively, we demonstrate that the newly identified O-glycosylated residues Thr 222 and Thr 225 of Ncr1 are essential for its activity, *in vitro* and *in vivo*.

### The novel O-linked glycosylations of Ncr1 are important for its function in primary NK cells

**Figure 7:**
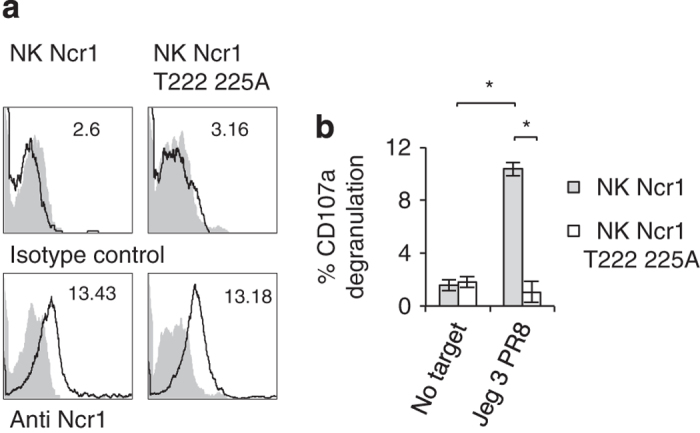
The novel O-linked glycosylations of Ncr1 are important for its function in primary human NK cells. (**a**) FACS staining of Ncr1 and Ncr1 T222 225A expression in human NK cells. The upper histogram shows the staining of untransfected cells and the lower histograms depict the staining of the transfected cells. The filled gray histograms represent staining with isotype control mAb. The MFIs are indicated. (**b**) % CD107a expression on the indicated NK transfectants in the presence of PR8-treated Jeg3 cells. The effector to target ratio was 1:1. Values are shown as mean±s.e.m. *P<0.05. The figure represents data collected from three independent experiments.

We demonstrated above using fusion proteins, HPLC and reporter assays that Ncr1 carries O-glycosylations essential for its function. O-glycosylations might vary between different cells and thus ideally it would be important to demonstrate that the newly identified putative O-glycosylations function in primary mouse NK cells. We initially tried expressing the WT Ncr1 and the mutated Ncr1 proteins in the mouse NK cells derived from Ncr1 knockout mice (Ncr1^*gfp/gfp*^) [2]. Despite our best efforts and although we employed several methods of gene insertion (including retro- and lentiviral infection), we were unsuccessful. Importantly, we were able to express the Ncr1 and mutated Ncr1 in IL2-activated human primary NK cells, using lentiviral infection (see detailed protocol in the Materials and Methods section). The expression levels of Ncr1 mutated at the O-linked residues and of WT Ncr1 were similar (Figure 7a). In agreement with our above results, the Ncr1 protein mutated in all N-glycosylated positions could not be expressed in IL2-activated primary human NK cells.

As human NK cells express receptors that bind and are activated by influenza virus HA (NKp44 and NKp46) [1, 16, 17, 20], we had to block these interactions using blocking mAbs. Next, in order to determine whether the O-glycosylated residues of Ncr1 are essential for its influenza virus recognition, we expressed the WT Ncr1 or Ncr1 T222 225A proteins in primary bulk IL2-activated human NK cells, blocked NKp44 and NKp46 with anti-NKp44- and anti-NKp46-blocking mAbs and incubated the Ncr1 and Ncr1 T222 225A transduced NK cells with PR8-infected Jeg3 cells. We used Jeg3 cells here because these are human cells that do not express any known ligands for NK-activating receptors and are therefore minimally killed in the absence of influenza. NK cell cytotoxicity was assayed by CD107a degranulation assays. Importantly, we observed significant degranulation with the WT Ncr1-expressing human NK cells and practically no degranulation with the T222 225A mutant (Figure 7b).

## Discussion

Human NKp46 and murine Ncr1 share many structural similarities. However, although NKp46 is predicted to have two O-linked glycosylations and one N-linked glycosylation, the murine Ncr1 was predicted to have, only N-linked glycosylations at positions Asn 139, Asn 216 and Asn 238 [14]. Using the NetOGlyc 4.0 Server for mucin type O-GalNAc O-glycosylation [22], combined with HPLC analysis and mutants generation, we identified two putative previously uncharacterized Thr residues (Thr 222 and Thr 225) carrying O-linked glycosylations in Ncr1, which are essential for its function.

Ideally, it would be interesting to identify the exact glycan residues of Ncr1 isolated from mouse cells by mass spectrometry-based methods. However, these experiments, using the technology at hand, would require sacrificing thousands of mice. Instead, we immunoprecipitated the endogenous Ncr1 protein from primary mouse NK cells and demonstrated that Ncr1 carries putative O-glycosylations. In addition, we expressed the Ncr1 and O-glycosylated mutant in primary human NK cells and demonstrated that the newly identified O-glycosylated residues of Ncr1 are essential for influenza recognition.

Influenza virus infection poses a severe health threat and a substantial economic burden to modern society [30, 31]. The several influenza virus pandemics outbreaks that had occurred in the last two centuries, stress the importance of studying and understanding the influenza virus activity and its interactions with the immune system.

In 2001, the influenza virus and Sendai virus’ HAs were discovered as ligands for the activating receptors NKp46 and NKp44 [20]. Sialic acids attached to O-glycosylated residues of NKp46 were shown to be critical for their interaction with HA [1, 20].

The sialic acid-based mechanism of NKp46 recognition of infected cells was probably developed by the NK cells to ensure the recognition of various influenza viruses. The influenza virus HA undergoes rapid and extensive changes to avoid detection by the immune system [32] and indeed around 10 000 different viral HA sequences exist in the data bank. However, the ability of viral HA to bind sialic acids is conserved [33]. NK cells utilize this property to kill the infected cells via the interaction between viral HA and the sialic acids residues expressed, as we show here, by O-glycosylated residues on Ncr1.

We demonstrated here that the newly identified putative O-glycosylated residues of Ncr1 are essential for its HA recognition. Remarkably, although the human NKp46 and mouse Ncr1 differ in their glycosylations, still residue T225 is conserved in humans and mice. Interestingly, however, in Ncr1, an additional residue (T222) is important for Ncr1 recognition and function. We think that the proximity between the 222 and 225 glycosylations probably hints at some redundancy. It is common for a change in one glycosylation site to affect the glycosylation at other positions in the protein. Indeed, we demonstrated that the disruption of one Ncr1 Thr residue or both residues together produced more or less the same effects. With this regard, the T222 and T225 positions of Ncr1 are located in the stalk region, where two of the protein’s N-linked glycosylations are also located. Thus, it might be that the N-glycosylations of these proximal residues are also affected. However, even if this is the case, it has no role in Ncr1 function, as mutating all N-linked glycosylated residues of Ncr1 did not affect its recognition, binding or function.

Mutating each of the newly identified putative O-glycosylated residues and of both residues together impaired not only the Ncr1 recognition of HA but also the recognition of various mouse and human tumors. As the NKp46/Ncr1 cellular ligands are unknown, it is quite difficult to directly test these interactions. Nevertheless, our results suggest that the putative O-glycosylations of Ncr1 are involved in the recognition of many mouse and human tumors. NKp46 and Ncr1 are also similar in that sense as the O-glycosylations of NKp46 were also shown to be important for its tumor recognition [1].

As both O-glycosylated putative positions of Ncr1 reside in the unstructured stalk domain, it is very unlikely that mutating these residues disrupted the Ncr1 structure. To further exclude this unlikely possibility, we produced eight new anti-Ncr1 mAbs and demonstrated that all recognized the WT and mutated Ncr1 proteins. In addition, when expressed in BW cells the WT and O-linked mutated Ncr1 proteins were equally well expressed on the cell surface and were equally recognized by anti-Ncr1 mAbs. Furthermore, equal expression levels of the WT and the O-glycosylation mutants were detected on the surface of primary human NK cells.

Thus, we do not think that mutating the O-glucosylated residues of Ncr1 had an effect on the Ncr1 protein structure.

Interestingly, although unstructured, the stalk domains have an important role in the recognition of several targets by NCRs members. In NKp44 and NKp46, sialic acids moieties in the stalk domain interact with viral HAs [1, 18, 19, 20]. NKp30 was also shown to interact with its targets in a stalk domain-glycosylations-mediated manner [34].

The Ncr1 was cloned almost two decade ago [14] and since then it was demonstrated to be critical in many pathological conditions [1–8, 10, 11, 16–20, 35]. However, the identity of the cellular ligand/s recognized by Ncr1 is still unknown. We demonstrate here that putative O-glycosylations of Ncr1 are important for all interactions. It is thus possible that similarly to HAs (the viral ligands of Ncr1), the tumor ligands or co-ligands recognized by Ncr1 might be lectins.

## Materials and Methods

### Generation of Ig fusion proteins, cell transfectants and TM treatment

Fusion proteins were generated in HEK293T cells, as previously described [20]. In short, the extracellular parts of Ncr1, the HA of PR8 influenza virus, NKp44 and NKp46 were amplified by PCR and cloned in frame with human IgG1.

Mutations in the Ncr1 protein were generated by PCR. For the generation of the lentiviral vectors, the Ncr1 proteins were cloned and the various mutations were generated by PCR. TM was used at 5 μg ml when relevant. For the generation of the BW reporter cells, the extracellular parts of the Ncr1 and various Ncr1 mutants described above were fused to the mouse CD3 zeta chain. The various Ncr1 zeta constructs were expressed in a lentiviral vector and transduced in BW cells. The BW assays were performed as previously described [20]. In brief, BW cells expressing the various Ncr1 receptors fused to the mouse zeta chain were incubated at a ratio of 1:1 with target cells at 37 °C and 5% CO_2_. Following 48 h of incubation, the supernatants were collected, and IL2 levels were measured using standard enzyme-linked immunosorbent assay.

### Generation of anti-mNcr1 mAbs and blocking of Ig fusion proteins binding

The various anti-mNcr1 mAbs used in this study were generated by immunizing Ncr1^*gfp/gfp*^Balb/c mice [2, 18] with the Ncr1 Ig fusion protein that we have generated. Biotynilation of Ncr1 Ig was performed using EZ-Link Sulfo-NHS-SS-Biotin (Thermo Scientific, Rockford, IL, USA) according to the manufacturer’s instruction. For blocking the staining by the biotynilated Ncr1 Ig, each of the WT and mutated fusion proteins used in this study were incubated with the untreated or PR8-treated EL4 cells for 2 h. Next, the biotinylated Ncr1 Ig was added to the cells incubated with the various fusion proteins, left on ice for an additional hour and then the cells were washed and stained with a streptavidin-conjugated secondary mAb.

### Immunoprecipitation and western blotting

Immunoprecipitation of Ncr1 was performed following standard immunoprecipitation protocol, using agarose A/G beads and the anti-Ncr1 mNcr1.6 mAb. For western blotting, the precipitated proteins were run on a 10% SDS-PAGE gel, transferred and stained with anti-mNcr1. For the western blotting with lectins, the various fusion proteins (5 μg) were run on 10% SDS-PAGE gel, transferred to a nitrocellulose membrane (Tamar, Mevaseret Zion, Israel) and blotted with biotin-conjugated lectins (Jacalin, wheat germ agglutinin, Maackia amurensis lectin II and SNA, all from Vector labs, Burlingame, CA, USA) or with biotin-conjugated HA Ig. The staining was visualized using a streptavidin HRP secondary reagent and EZ ECL substrate (Biological Industries, Beit-Haemek, Israel).

### Preparation of the fusion proteins for sugar content analysis and HPLC

For O-glycosylation analysis, 250 μg purified Ncr1 Ig and Ncr1 T222 225A Ig were reduced with 10 mM dithiothreitol for 10 min at 70 °C, before alkylation with 55 mM iodoacetamide for 30 min at room temperature (protected from light). The Ncr1 Ig Ncr1 T222A Ig, Ncr1 T225a Ig and Ncr1 T222 225A Ig N-linked glycans were released in solution with PNGase F.

For O-glycan release, the de-N-glycosylated glycoproteins were lyophilized before hydrazinolysis. Samples were incubated with anhydrous hydrazine for 6 h at 60 °C to release the O-linked glycan (glycan hydrazinolysis kit, catalog no. GK50202; Glyco/Prozyme (ProZyme, Inc., Hayward, CA, USA)). Excess hydrazine was removed by evaporation, and the glycans were re-N-acetylated with acetic anhydride in a saturated solution of sodium bicarbonate. Peptides were removed by descending paper chromatography on prewashed Whatmann 3-mm chromatography paper in butanol/ethanol/water (8:2:1 (vol/vol)) for 70 h. Glycans were recovered from the paper (1–3 cm from origin) by washing with 1.5 ml of water. A rotary evaporator was used to concentrate the samples, which were then stored at 20 °C before labeling with 2AB (2-aminobenzamide).

Exoglycosidase digestion of the sialic acid was performed on the released, 2AB-labeled glycan solution containing *Arthrobacter ureafaciens* sialidase (sialidase A, cleaves both 2,3 and 2,6 sialic acid residues; ABS, Glyco/Prozyme). Normal phase HPLC was performed with the low-salt buffer system using a 4.6- by 250-mm GlycoSep N column (OGS; Waters, Milford, MA, USA). The solvents used were buffer B (50 mM ammonium formate, pH 4.4) and buffer C (acetonitrile). The glycans were eluted by linear gradient with buffer B, with initial conditions of 20% buffer B at a flow rate of 0.4 ml min. The concentration of buffer B was changed from 35 to 53% over 132 min and then from 53 to 100% over the next 3 min, with a constant flow rate. The column was washed with 100% buffer B for 5 min at a flow rate of 1 ml min before re-equilibration in the initial solvent system. Fluorescence was measured at 420 nm with an excitation of 330 nm (with 16-nm bandwidths).

### Surface plasmon resonance

Surface plasmon resonance assays were performed using Biacore T100 biosensor (GE Healthcare, Uppsala, Sweden). WT and mutated Ncr1 were dissolved in Biacore immobilization buffer (10 mM sodium acetate, pH 4.0) to 30 nM and then coupled to CM5 Series S chip (GE Healthcare) using manufacturer-recommended amine-coupling protocol to a level of 500RU. Recombinant H1 HA wth His Tag form Influenza A/Brisbane/59/2007 H1N1 56 FR-69 (IRR, CDC) was dissolved in the HBS-EP+ buffer (10 mM HEPES pH 7.3, 150 mM NaCl, 3 mM EDTA pH8 and 0.05% Tween20) and was injected in concentrations: 0.78, 1.56, 3.12, 6.25, 12.5, 25, 50 and 100 nM using 50 μl min flow rate; 100 s. Dissociation was measured for 120 s. The surface was regenerated by a single 30-s pulse of 10 mM NaOH.

### Mice and influenza virus

All experiments were performed using 4–5-week-old C57BL/6 mice. The generation of the Ncr1^*gfp/gfp*^ mouse was previously described [2]. All the experiments were performed in a specific pathogen-free unit of the Hebrew University Medical School (Ein-Kerem, Jerusalem) in accordance with the guidelines of the ethics committee (MD-12-13471-5). Propagation of the human influenza virus A/Puerto Rico/8/34 H1N1 (PR8) was performed as previously described [18].

### Expression of Ncr1 in primary human NK cells and CD107a assay

The full-length Ncr1 and T222 225A Ncr1 were each cloned into a lentiviral vector (pHAGE-DsRED(−)-eGFP(+), which also contains green fluorescent protein (GFP) [36]). 293T cells (3.5×10^5^ per well) were seeded in 15 six-well plates in 2 ml DMEM a day prior transfection. The WT or O-glycosylated mutant of Ncr1 vectors, a plasmid encoding the lentiviral Gag/Pol, and a plasmid encoding the VSV-G were transfected in the 293T cells using Mirus reagent (TransIT-LT1, Mirus, Madison, WI, USA), according to the manufacturer instructions. Forty-eight hours following transfection, 180 ml of medium containing the lenti viruses was collected. The medium was then filtered using 0.2-μm filters and the lenti viruses were used to infect primary IL2-activated bulk human NK cells. The human NK cells used in this study were obtained from the blood of healthy volunteers. The institutional Helsinki committee of Hadassah approved the study (Helsinki number 0030-12-HMO). All subjects provided a written informed consent. Peripheral blood mononuclear cells (PBMCs) were purified from heparinized blood by the centrifugation on LymphoprepTM (StemCells Technologies, Vancouver, BC, Canada). NK cells were isolated using the EasySepTM human NK cell enrichment kit (StemCells Technologies). Activated NK cells were generated by culturing isolated NK cells together with irradiated feeder cells (2.5×10^4^ allogeneic PBMCs from two donors and 5×10^3^ 8866 B cells in each well) and 20 μg ml PHA (Roche, Rehovot, Israel). Both PBMCs and 8866 B cells were irradiated in 6000 rad before seeding in 96-well U-bottom plates. The cultures were maintained in DMEM:F-12 Nutrient Mix (Sigma Aldrich, Rehovot, Israel; 70:30), 10% human serum (Sigma Aldrich), 2 mM glutamine (Biological Industries; BI, Beit-Haemek, Israel), 1 mM sodium pyruvate (BI), 1× nonessential amino acids (BI), 100 U ml penicillin (BI), 0.1 mg ml streptomycin (BI) and 500 U ml rhIL-2 (Peprotech, Rehovot, Israel). NK cells were stained with anti-CD56 phycoerythrin (BD Biosciences, San Jose, CA, USA) and anti-CD3 allophycocyanin (Biolegend, San Diego, CA, USA) to confirm NK purity after isolation and following activation. A week later, irradiated feeder cells (2.5×10^4^ allogeneic PBMC from two donors and 5×10^3^ 8866 B cells in each well) were added.

Two hundred NK cells were seeded per well of each of 18 96-well plates in 100 μl of the filtered virus-containing DMEM medium supplemented with 2 mM glutamine (BI), 1 mM sodium pyruvate (BI), 1× nonessential amino acids (BI), 100 U ml penicillin (BI) and 0.1 mg ml streptomycin (BI). Then, the plates were centrifuged for 2 h at 28 °C, 648*g*. On the next day (24 h following infection) 100 μl were added of the DMEM:F-12 Nutrient Mix (70:30), 10% human serum (Sigma Aldrich), 2 mM glutamine (Biological industries; BI), 1 mM sodium pyruvate (BI), 1× nonessential amino acids (BI), 100 U ml penicillin (BI), 0.1 mg ml streptomycin (BI) and 500 U ml rhIL-2 (Peprotech). On the next day (48 h following infection), the cells were collected and sorted using SH800Z Cell Sorter Sony Biotechnology Inc. (Weybridge, Surrey, UK) based on GFP expression. We then verified that Ncr1 is expressed using an allophycocyanin-conjugated anti-mNKp46/Ncr1 (29A1.4 (Rat IgG2a, κ; Biolegend) mAb.

For CD107a degranulation assays of the Ncr1-expressing human NK cells, 1000 NK cells per well were first pre-blocked with 0.2 μg of each of the blocking mAbs anti-NKp44 (clone p44-8 cat #325104) and anti-NKp46 (clone 9E2 cat #331904; Bioledgend). Then, the transfected NK cells were incubated with PR8-treated Jeg3 cells in a ratio of 1:1 in the presence of 0.1 μg allophycocyanin-conjugated CD107a mAb (Biotest, Ness Ziona, Israel) for 2 h at 37 °C. CD107a levels on the NK cells were determined by flow cytometry.

### Statistical analysis

Analysis of variance was used to identify significant group differences.

## References

[bib1] Arnon TI , Achdout H , Lieberman N et al. The mechanisms controlling the recognition of tumor- and virus-infected cells by NKp46. Blood 2004; 103: 664–672. 14504081 10.1182/blood-2003-05-1716

[bib2] Gazit R , Gruda R , Elboim M et al. Lethal influenza infection in the absence of the natural killer cell receptor gene Ncr1. Nat Immunol 2006; 7: 517–523. 16565719 10.1038/ni1322

[bib3] Jarahian M , Fiedler M , Cohnen A et al. Modulation of NKp30- and NKp46-mediated natural killer cell responses by poxviral hemagglutinin. PLoS Pathogens 2011; 7: e1002195. 21901096 10.1371/journal.ppat.1002195 PMC3161980

[bib4] Elboim M , Gazit R , Gur C , Ghadially H , Betser-Cohen G , Mandelboim O . Tumor immunoediting by NKp46. J Immunol 2010; 184: 5637–5644. 20404273 10.4049/jimmunol.0901644

[bib5] Glasner A , Ghadially H , Gur C et al. Recognition and prevention of tumor metastasis by the NK receptor NKp46/NCR1. J Immunol 2012; 188: 2509–2515. 22308311 10.4049/jimmunol.1102461

[bib6] Halfteck GG , Elboim M , Gur C , Achdout H , Ghadially H , Mandelboim O . Enhanced in vivo growth of lymphoma tumors in the absence of the NK-activating receptor NKp46/NCR1. J Immunol 2009; 182: 2221–2230. 19201876 10.4049/jimmunol.0801878

[bib7] Lakshmikanth T , Burke S , Ali TH et al. NCRs and DNAM-1 mediate NK cell recognition and lysis of human and mouse melanoma cell lines *in vitro* and *in vivo*. J Clin Invest 2009; 119: 1251–1263. 19349689 10.1172/JCI36022 PMC2673866

[bib8] Gur C , Enk J , Kassem SA et al. Recognition and Killing of Human and Murine Pancreatic {beta} Cells by the NK Receptor NKp46. J Immunol 2011; 187: 3096–3103. 21849674 10.4049/jimmunol.1101269

[bib9] Gur C , Enk J , Weitman E et al. The expression of the beta cell-derived autoimmune ligand for the killer receptor nkp46 is attenuated in type 2 diabetes. PloS one 2013; 8: e74033. 24009765 10.1371/journal.pone.0074033 PMC3757008

[bib10] Chaushu S , Wilensky A , Gur C et al. Direct recognition of Fusobacterium nucleatum by the NK cell natural cytotoxicity receptor NKp46 aggravates periodontal disease. PLoS Pathog 2012; 8: e1002601. 22457623 10.1371/journal.ppat.1002601 PMC3310798

[bib11] Gur C , Coppenhagen-Glazer S , Rosenberg S et al. Natural killer cell-mediated host defense against uropathogenic *E. coli* is counteracted by bacterial hemolysinA-dependent killing of NK cells. Cell Host Microbe 2013; 14: 664–674. 24331464 10.1016/j.chom.2013.11.004 PMC3868942

[bib12] Gur C , Ibrahim Y , Isaacson B et al. Binding of the Fap2 Protein of Fusobacterium nucleatum to Human Inhibitory Receptor TIGIT Protects Tumors from Immune Cell Attack. Immunity 2015; 42: 344–355. 25680274 10.1016/j.immuni.2015.01.010 PMC4361732

[bib13] Moretta A , Bottino C , Vitale M et al. Activating receptors and coreceptors involved in human natural killer cell-mediated cytolysis. Ann Rev Immunol 2001; 19: 197–223. 11244035 10.1146/annurev.immunol.19.1.197

[bib14] Biassoni R , Pessino A , Bottino C , Pende D , Moretta L , Moretta A . The murine homologue of the human NKp46, a triggering receptor involved in the induction of natural cytotoxicity. Eur J Immunol 1999; 29: 1014–1020. 10092106 10.1002/(SICI)1521-4141(199903)29:03<1014::AID-IMMU1014>3.0.CO;2-O

[bib15] Ghadially H , Horani A , Glasner A et al. NKp46 regulates allergic responses. Eur J Immunol 2013; 43: 3006–3016. 23878025 10.1002/eji.201343388 PMC3867659

[bib16] Bar-On Y , Glasner A , Meningher T et al. Neuraminidase-mediated, NKp46-dependent immune-evasion mechanism of influenza viruses. Cell Rep 2013; 3: 1044–1050. 23602571 10.1016/j.celrep.2013.03.034 PMC3863986

[bib17] Bar-On Y , Seidel E , Tsukerman P , Mandelboim M , Mandelboim O . Influenza virus uses its neuraminidase protein to evade the recognition of two activating NK cell receptors. J Infec Dis 2014; 210: 410–418. 24532603 10.1093/infdis/jiu094 PMC4074429

[bib18] Glasner A , Zurunic A , Meningher T et al. Elucidating the mechanisms of influenza virus recognition by Ncr1. PLoS ONE 2012; 7: e36837. 22615821 10.1371/journal.pone.0036837 PMC3352933

[bib19] Jarahian M , Watzl C , Fournier P et al. Activation of natural killer cells by newcastle disease virus hemagglutinin-neuraminidase. J Virol 2009; 83: 8108–8121. 19515783 10.1128/JVI.00211-09 PMC2715740

[bib20] Mandelboim O , Lieberman N , Lev M et al. Recognition of haemagglutinins on virus-infected cells by NKp46 activates lysis by human NK cells. Nature 2001; 409: 1055–1060. 11234016 10.1038/35059110

[bib21] Pessino A , Sivori S , Bottino C et al. Molecular cloning of NKp46: a novel member of the immunoglobulin superfamily involved in triggering of natural cytotoxicity. J Exp Med 1998; 188: 953–960. 9730896 10.1084/jem.188.5.953 PMC3207313

[bib22] Steentoft C , Vakhrushev SY , Joshi HJ et al. Precision mapping of the human O-GalNAc glycoproteome through SimpleCell technology. EMBO J 2013; 32: 1478–1488. 23584533 10.1038/emboj.2013.79 PMC3655468

[bib23] Roque-Barreira MC , Campos-Neto A . Jacalin: an IgA-binding lectin. J Immunol 1985; 134: 1740–1743. 3871459

[bib24] Monsigny M , Roche AC , Sene C , Maget-Dana R , Delmotte F . Sugar-lectin interactions: how does wheat-germ agglutinin bind sialoglycoconjugates? Eur J Biochem/FEBS 1980; 104: 147–153. 10.1111/j.1432-1033.1980.tb04410.x 6892800

[bib25] Cantoni C , Ponassi M , Biassoni R et al. The three-dimensional structure of the human NK cell receptor NKp44, a triggering partner in natural cytotoxicity. Structure 2003; 11: 725–734. 12791260 10.1016/s0969-2126(03)00095-9

[bib26] Foster CE , Colonna M , Sun PD . Crystal structure of the human natural killer (NK) cell activating receptor NKp46 reveals structural relationship to other leukocyte receptor complex immunoreceptors. J Biol Chem 2003; 278: 46081–46086. 12960161 10.1074/jbc.M308491200

[bib27] Joyce MG , Tran P , Zhuravleva MA , Jaw J , Colonna M , Sun PD . Crystal structure of human natural cytotoxicity receptor NKp30 and identification of its ligand binding site. Proc Natl Acad Sci USA 2011; 108: 6223–6228. 21444796 10.1073/pnas.1100622108 PMC3076882

[bib28] Li Y , Wang Q , Mariuzza RA . Structure of the human activating natural cytotoxicity receptor NKp30 bound to its tumor cell ligand B7-H6. J Exp Med 2011; 208: 703–714. 21422170 10.1084/jem.20102548 PMC3135353

[bib29] Sata T , Roth J , Zuber C , Stamm B , Heitz PU . Expression of alpha 2,6-linked sialic acid residues in neoplastic but not in normal human colonic mucosa. A lectin-gold cytochemical study with Sambucus nigra and Maackia amurensis lectins. Am J Pathol 1991; 139: 1435–1448. 1661075 PMC1886452

[bib30] Horimoto T , Kawaoka Y . Influenza: lessons from past pandemics, warnings from current incidents. Nat Rev Microbiol 2005; 3: 591–600. 16064053 10.1038/nrmicro1208

[bib31] Neumann G , Noda T , Kawaoka Y . Emergence and pandemic potential of swine-origin H1N1 influenza virus. Nature 2009; 459: 931–939. 19525932 10.1038/nature08157 PMC2873852

[bib32] Hensley SE , Das SR , Bailey AL et al. Hemagglutinin receptor binding avidity drives influenza A virus antigenic drift. Science 2009; 326: 734–736. 19900932 10.1126/science.1178258 PMC2784927

[bib33] Skehel JJ , Wiley DC . Receptor binding and membrane fusion in virus entry: the influenza hemagglutinin. Ann Rev Biochem 2000; 69: 531–569. 10966468 10.1146/annurev.biochem.69.1.531

[bib34] Hartmann J , Tran TV , Kaudeer J et al. The stalk domain and the glycosylation status of the activating natural killer cell receptor NKp30 are important for ligand binding. J Biol Chem 2012; 287: 31527–31539. 22807449 10.1074/jbc.M111.304238 PMC3438985

[bib35] Gur C , Doron S , Kfir-Erenfeld S et al. NKp46-mediated killing of human and mouse hepatic stellate cells attenuates liver fibrosis. Gut 2012; 61: 885–893. 22198715 10.1136/gutjnl-2011-301400

[bib36] Yamin R , Kaynan NS , Glasner A et al. The viral KSHV chemokine vMIP-II inhibits the migration of Naive and activated human NK cells by antagonizing two distinct chemokine receptors. PLoS Pathog 2013; 9: e1003568. 23966863 10.1371/journal.ppat.1003568 PMC3744409

