# Supplementary material for: Identification of putative novel O-glycosylations in the NK killer receptor Ncr1 essential for its activity
Source: Cell Discov. 2015 Dec 22;1:15036–. doi: 10.1038/celldisc.2015.36 (PMC4860851; doi:10.1038/celldisc.2015.36)
Supplement: Supplementary Information [file celldisc201536-s1.pdf]

Supp 1

Signal Peptide MLPTLTALLCLGLCLSQ

Ig Domain I

RINTEKETLPKPIIWAKPSIMVTNGNSVNIWCQGAQSASEYQLYFEGSFFALERPKPSR
SMNKVRFFISQMTSHTAGIYTCFYQSGELWSKSSNPLKLVVTGL YDTPNLWVY

Ig Domain II

PRPEVTLGENVTFFCQLKTATSKFFLLKERGSNHIQNKYGNIQAEFPMGPVTRAHRG
TYRCFGSYNDYAWSFPSEPVTLLIT

Stalk Region GGVENSSLAPTDPTSSLDYWEFDLSTNES
GLQKDSAFWDHTTQN

Transmembrane LIRIGLACIILITLVWLL

Cytoplasmic

TEDWLSKRKDHEEANRLTNWECRRRWR MQHYFEEEQRNAISMMELKATPGAL

N139

N216

N238

T222

T225

Supp 2

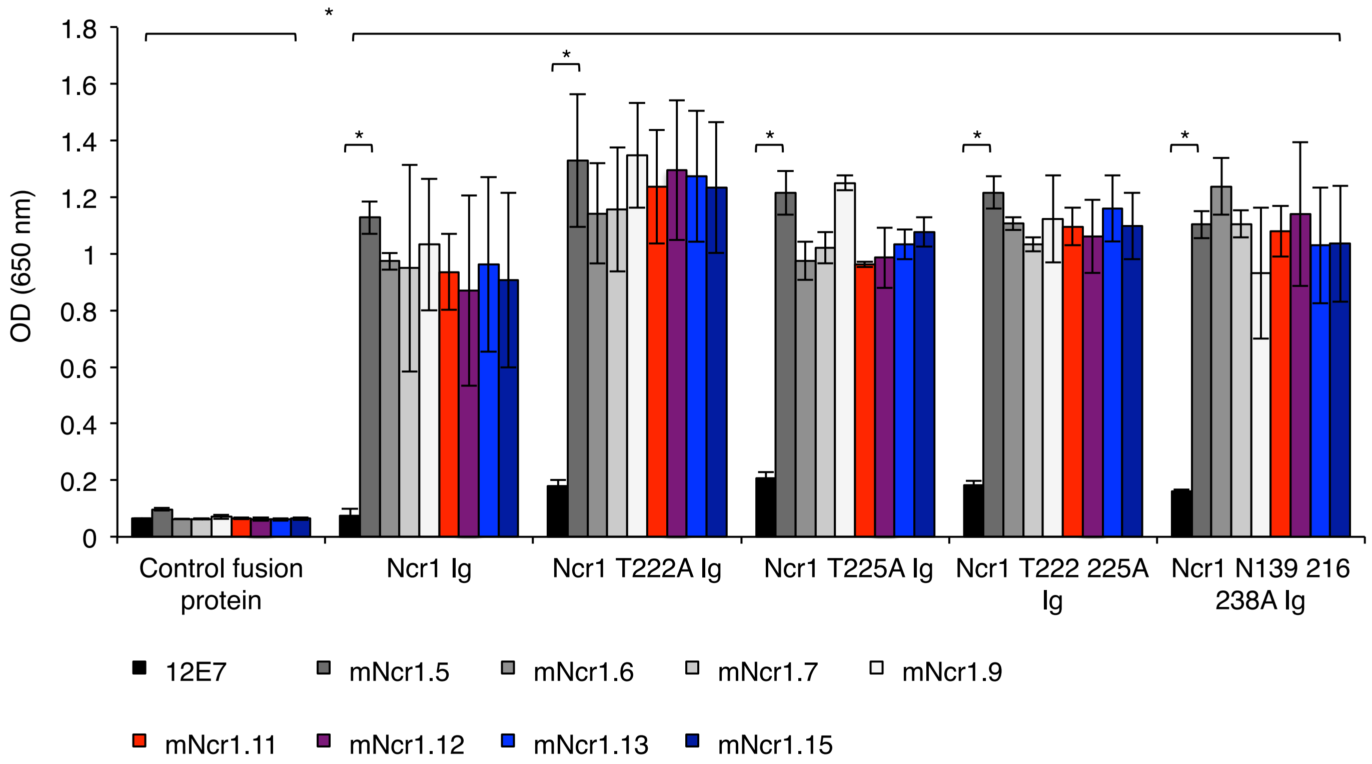

OD (650 nm)
1.8
1.6
1.4
1.2
1
0.8
0.6
0.4
0.2
0
*
*
*
*
*
*
Control fusion protein
Ncr1 Ig
Ncr1 T222A Ig
Ncr1 T225A Ig
Ncr1 T222 225A Ig
Ncr1 N139 216 238A Ig
12E7
mNcr1.5
mNcr1.6
mNcr1.7
mNcr1.9
mNcr1.11
mNcr1.12
mNcr1.13
mNcr1.15

Supp 3

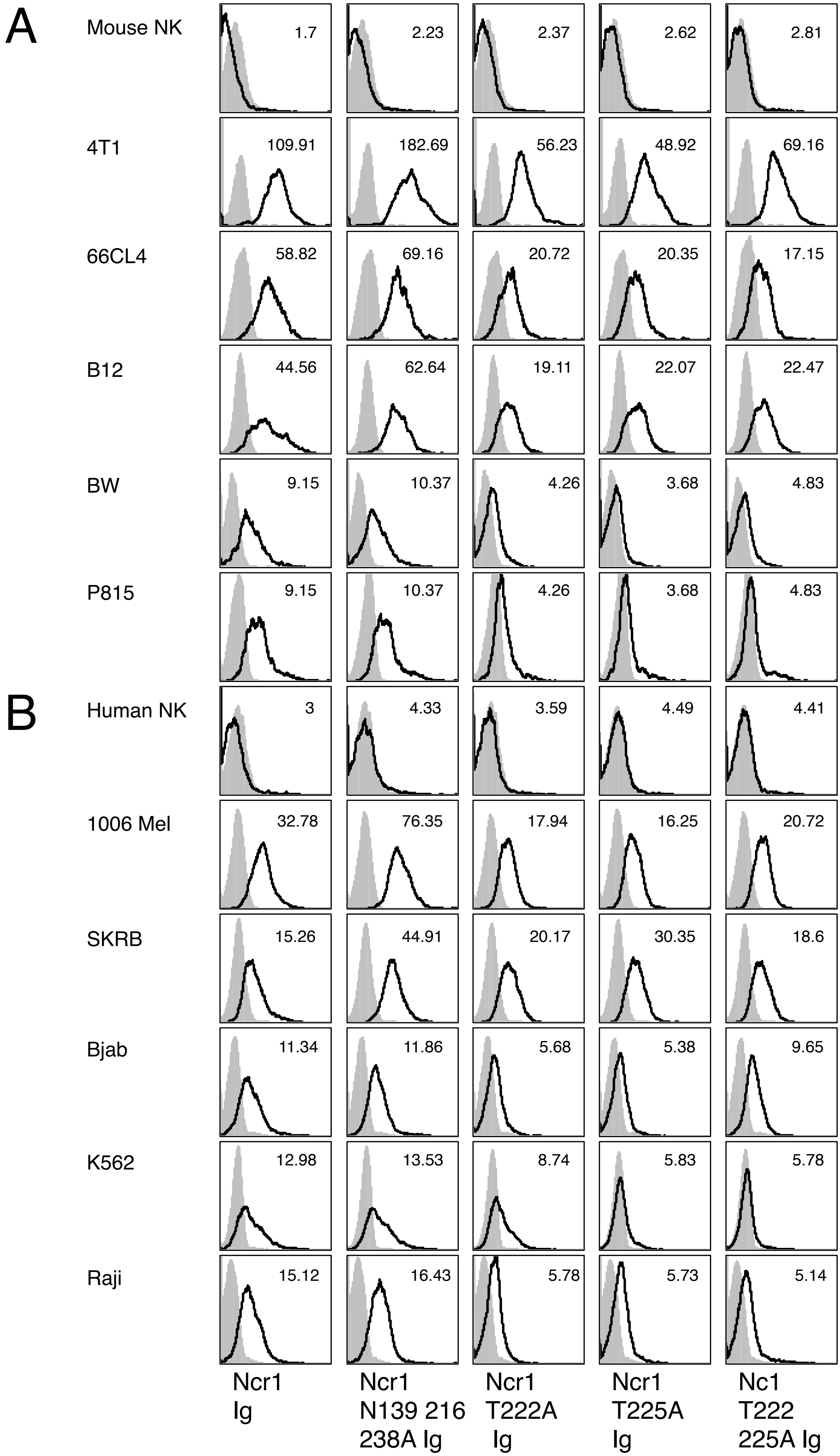
A
Mouse NK
1.7
2.23
2.37
2.62
2.81
4T1
109.91
182.69
56.23
48.92
69.16
66CL4
58.82
69.16
20.72
20.35
17.15
B12
44.56
62.64
19.11
22.07
22.47
BW
9.15
10.37
4.26
3.68
4.83
P815
9.15
10.37
4.26
3.68
4.83
B
Human NK
3
4.33
3.59
4.49
4.41
1006 Mel
32.78
76.35
17.94
16.25
20.72
SKRB
15.26
44.91
20.17
30.35
18.6
Bjab
11.34
11.86
5.68
5.38
9.65
K562
12.98
13.53
8.74
5.83
5.78
Raji
15.12
16.43
5.78
5.73
5.14
Ncr1 Ig
Ncr1 N139 216 238A Ig
Ncr1 T222A Ig
Ncr1 T225A Ig
Nc1 T222 225A Ig

Supplementary figure legends

Supplementary figure 1. Schematic representation of Ncr1 glycosylations
Schematic representation of the glycosylated amino acid residues of Ncr1. The different Ig-like domains, the stalk region, the transmembrane and cytoplasmic segments are shown. The predicted N-glycosylated residues at positions 139, 216 238 and the newly predicted O-linked glycosylations at position 222 and 225 are marked in color.

Supplementary figure 2. All WT and mutated Ncr1 fusion proteins are recognized by various anti mNcr1 mAbs.
ELISA assays of a control fusion protein and the various WT and mutated Ncr1 fusion proteins used in this study (indicated in the X axes) with a control mAb 12E7 and eight anti mNcr1 mAbs, as indicated below the figure. The figure is a representative of two independent experiments. Values are shown as mean ±SEM. *P<0.05.

Supplementary figure 3. WT and mutated Ncr1 recognition of tumors.
(A-B) FACS staining of various mouse (A) and human (B) cell lines and primary mouse (A) and human (B) NK cells, as indicated. The staining of the primary mouse NK cells is identical to the staining presented in figure 4A and is shown here again only for the sake of clarity. Staining was performed with various Ncr1 Ig fusion proteins (black line histograms). The gray filled histograms are the backgrounds secondary mAb staining. The staining is representative of at least three independent experiments. The MFIs are indicated.
